# An Unusual Vertical T12 Fracture Extending into the T12-L1 Disc in a Patient with Diffuse Idiopathic Spondylotic Hyperostosis: A Case Study Using Vertebroplasty to Stabilize the Fracture

**DOI:** 10.7759/cureus.4477

**Published:** 2019-04-16

**Authors:** Jason Hartman, Michelle Granville, Aldo Berti, Robert E Jacobson

**Affiliations:** 1 Pain Medicine, Larkin Community Hospital, Miami, USA; 2 Neurological Surgery, University of Miami Hospital, Miami, USA; 3 Neurological Surgery, Miami Neurosurgical Center, Miami, USA

**Keywords:** progressive vertebral fracture, vacuum phenomenon, vertebroplasty, vertical fracture, osteophyte fracture

## Abstract

Osteoporotic spinal fractures are seen above previous spinal instrumentation and also found in patients with diffuse idiopathic spondylotic hyperostosis (DISH). In both situations, there is marked spinal rigidity with a limited mobile spinal section that is vulnerable to motion and subsequent fracture with no or minimal trauma especially when there is concurrent osteoporosis. This is an unusual case where the patient developed a vertical anterior avulsion type fracture of T12 through a large bridging spondylotic ventral spur of bone, resulting in severe positional pain above a previous lumbar instrumented fusion. While being managed conservatively with bracing, sequential follow-up magnetic resonance imaging (MRI) and computed tomography (CT) scans showed progressive development of vacuum changes, both in the linear fracture and the adjacent intra-discal space. Vacuum changes are a strong radiologic sign of spinal instability. Because of age and not wanting to undergo further extensive fusion, he was treated with intra-discal and transpedicular placement of bone cement with the resolution of his pain.

## Introduction

Development of fractures at levels above previous lumbar spinal instrumentation is found in osteoporotic patients. This has been attributed to the rigidity of the instrumented lumbar section resulting in associated load shifting and then fracture [1]. Patients with large bridging ventral vertebral spurs and those with diffuse idiopathic skeletal hyperostosis (DISH) also develop spinal rigidity and are vulnerable to segmental spinal fractures. These fractures characteristically occur with minor trauma or just when positioned for anesthesia [2-3]. Avulsion fractures of the anterior lumbar vertebrae are uncommon but usually associated with hyper-extension trauma [4]. This is an unusual case involving a mixture of all of these factors involving an unusual vertical T12 fracture associated with an avulsed fracture of the ventral vertebral spur in an 80-year-old osteoporotic patient with previous lumbar fusion extending from S1 to L2, the level below the fracture. The fracture at T12, the T12-L1 interspace, as well as the L1-L2 interspace developed progressive worsening radiologic vacuum sign changes, a sign of spinal instability [5-7]. These vacuum changes progressed concurrently with the worsening localized pain which led to the concept of using bone cement to stabilize the fracture.

## Case presentation

**Figure 1:**
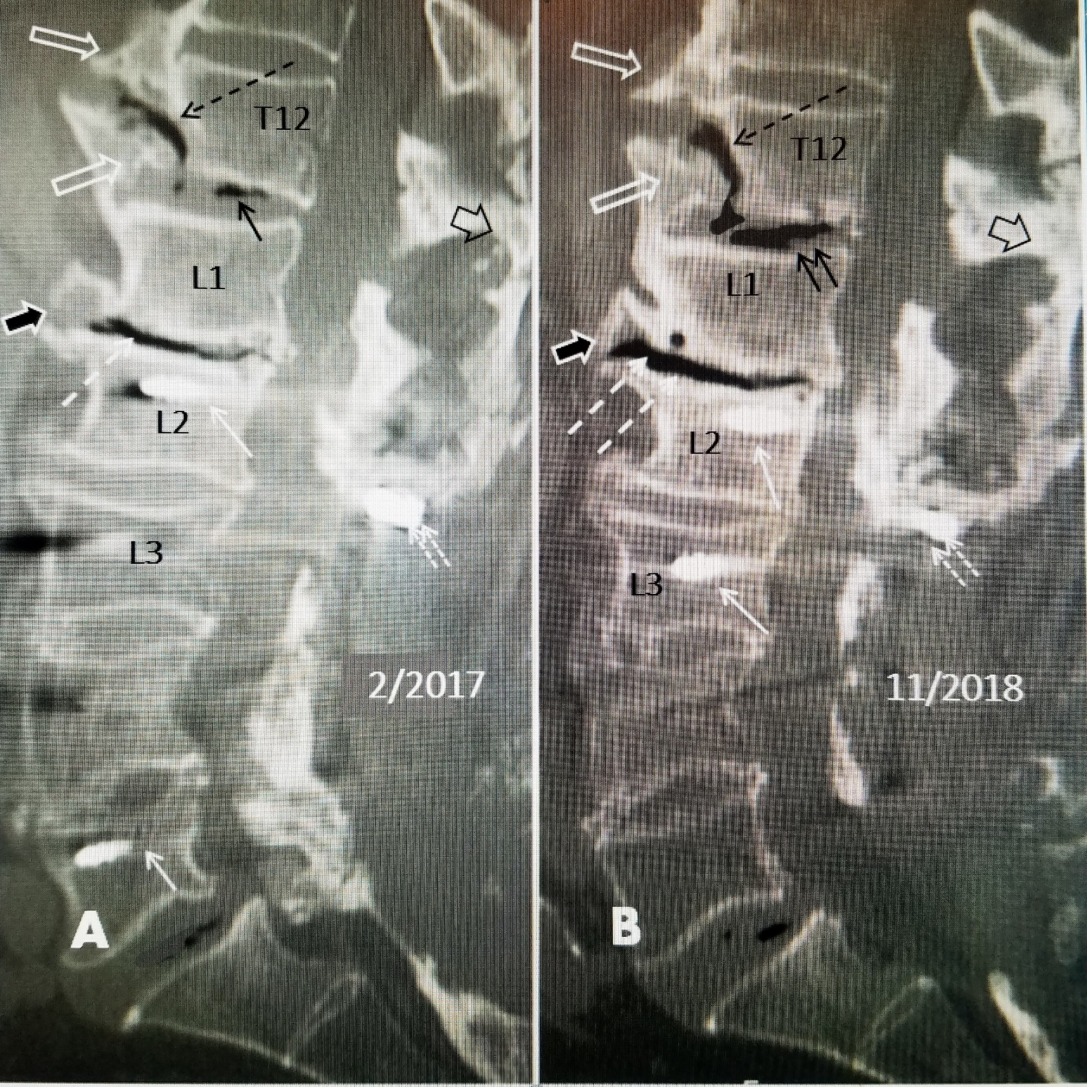
Lumbar and lower thoracic sagittal computerized tomography (CT) scan from February 2017 and November 2018 showing increasing vacuum changes at T12-L1 extending into the vertical T12 fracture A: Sagittal CT scan from February 2017 showing an anterior vertical avulsion fracture of inferior endplate of T12 (open white arrow) associated with vacuum signal change within the fracture (dashed black arrow). The T12-L1 disc space has vacuum changes (solid black arrow). There is also an osteophyte at L1-L2 ventral to the disc space (solid black arrow with white border). Vacuum change is also seen within the L1-L2 disc space (dashed white arrow). The supraspinous ligaments are calcified (open black arrow). Parts of pedicle screws are identified in the vertebral body (solid white arrows). B: Sagittal CT scan from November 2018, 21 months later, showing worsening vacuum changes compared to 2017. The avulsion fracture of the inferior endplate of T12 is unchanged in position (open white arrow) however the vacuum changes along the fracture line are more extensive (dashed black arrow). The osteophyte at L1-L2 (solid black arrow with white border) is unchanged. The vacuum change at T12-L1 disc space is more extensive (two small black arrows) as well as within the L1-L2 interspace (two dashed white arrows). The pedicle screws are marked at L1 (solid white arrow) and into the posterior bone (two small dashed white arrows). There is solid bone growth bridging the supraspinous ligament (open white arrow with black border) contributing to the spinal rigidity.

This 80-year-old male had a past medical history of colon resection with chemotherapy in 2000 and a stroke in 2005. In 2006, he had L3, L4 and L5 lumbar decompression and instrumentation for lumbar stenosis. He did well until 2009 when he developed low back and bilateral leg pain, more on the right. He had a magnetic resonance imaging (MRI) scan showing adjacent segment disease at L2-L3 with stenosis. He underwent a second lumbar surgery with an extension of the previous L3-L5 instrumentation to L2 with supplemental lateral mass bone fusion. He continued to complain of severe to moderate pain on a continual basis after which he elected to have an epidural neuromodulator placed in 2010. He developed an infection at the battery site and it was removed in 2011. From 2011 to 2018 he has multiple lumbar steroid injections for pain control and was taking opioids daily with only temporary relief. He then underwent a second implantation of a neuromodulator and his leg pain resolved, but shortly after that, he began complaining of upper low back and lower thoracic pain that was constant and different from his previous lumbar fusion pain. The area of pain was localized under fluoroscopy and found to be centered at the T12 and L1 spinal segment above the previous fusion and instrumentation at L2. When computerized tomography (CT) scans from early 2017 were compared to 2018 there were worsening vacuum changes within the T12 fracture as well as in the disc space at T12-L1. When reviewed with neuroradiology, it was felt that the vertical fracture line involving the anterior inferior one-third of T12 extended into the inferior endplate of T12, and connected to the T12-L1 interspace as well (Figure 1). 

**Figure 2:**
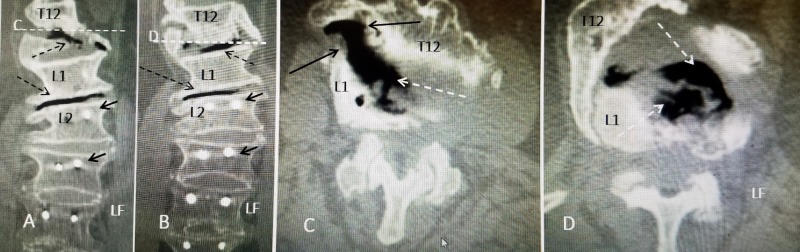
CT reconstruction comparing coronal and axial images showing extensive vacuum changes in the inter-vertebral disc space that are clearly extending into the avulsion fracture. A: Coronal CT reconstruction showing the level of axial slice 'C' (fine dashed white line with C). The vacuum change is seen within the avulsed fracture of T12 and T12-L1 and L1-L2 disc spaces (dashed black arrow). The pedicle screws at L2 and below are indicated (solid black arrow). Left is indicated (LF). B: Coronal axial CT showing axial slice 'D' (fine white line with D). The osteonecrosis and CT vacuum changes in T12-L1 and L1-L2 disc spaces are marked (dashed black arrow). The pedicle screws at L2 are indicated (solid black arrow). Left is indicated (LF). C: Axial CT made at the level of ventral osteophyte between T12 and L1 just below slice 'C'. There is vacuum signal change in the disc space (dashed white arrow) that extends both ventral and lateral into the fractured osteophyte between T12 (solid black arrow) and inferior edge of osteophyte at L1 (solid black arrow with white dots). D: Axial image showing vacuum change primarily within the disc space at T12 and L1 (dashed white arrow). Left is indicated (LF).

Detailed comparison of each coronal and axial CT reconstruction slice from 2018 clearly demonstrates the marked progression in the osteonecrosis along the T12 anterior fracture clearly connected to the T12-L1 interspace (Figure 2).

After detailed review of the different films with the patient, it was felt that stabilizing the fracture and T12-L1 disc space would be appropriate. He did not want to consider any further open instrumentation but agreed to percutaneous placement of cement along the fracture and into the disc space. It was also decided to use Cortoss^R^ cement (Stryker, Kalamazoo, Michigan, USA) which is both bioactive and has more fluid-like flow characteristics rather than denser polymethylmethacrylate bone cement (PMMA). It was felt this would allow the cement to flow better into the fractures and bone defects.

Technical steps of the procedure

**Figure 3:**
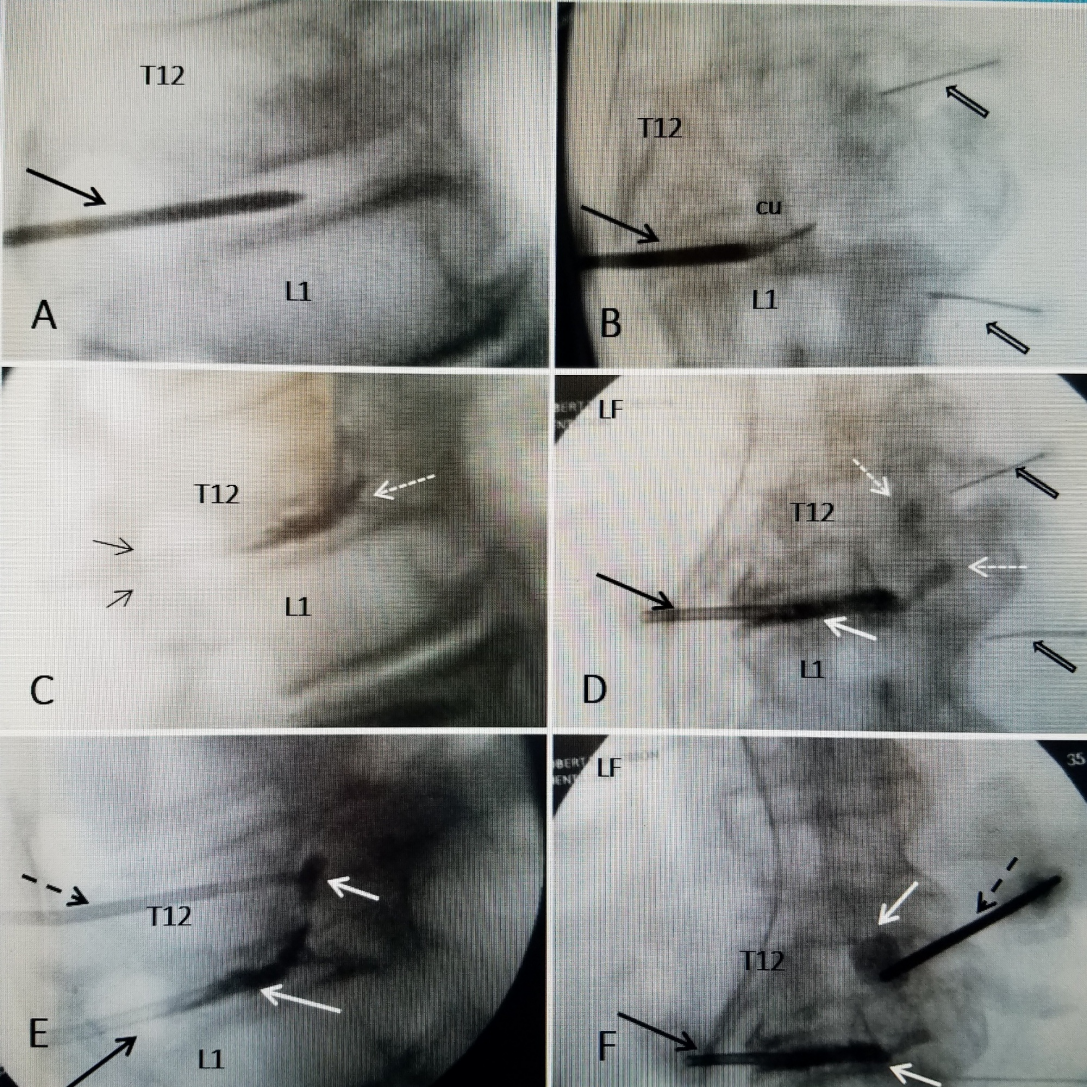
Sequential intra-operative fluoroscopic films A: Lateral fluoroscopy showing the initial 11 gauge vertebroplasty cannula (solid black arrow) within the T12-L1 disc space before contrast injection B: Anterior-posterior view showing curette (cu) advancing into the T12-L1 disc space toward the vertical fracture of T12 through the cannula (solid black arrow). The spinal needles mark the pedicles (wide black arrows). C: Initial injection of bone cement into the T12-L1 disc space (solid white arrow) through the cannula (black arrow). The cement is starting to move across the disc space toward the base of the fracture (dashed white arrow). D: Anterior-posterior (AP) view. The cannula (solid black arrow) and cement (solid white arrows) is close to the base of the vertical fracture of T12. The pedicles are marked with spinal needles (wide black arrows). E: Lateral view showing the second cannula through the pedicle of T12 vertebra (dashed black arrow). Cement has been injected into the vertical part of the fracture and actually flowed inferiorly toward the T12-L1 disc space connecting with the cement in the interspace (solid white arrows). F: AP view showing cannula through pedicle of T12 (dashed black arrow) with cement flowing into vertical avulsion fracture as well as interspace of T12-L1 (solid white arrows). The original cannula in the T12-L1 disc space can be seen (solid black arrow).

The procedure was divided into four different steps (Figure 3). The patient was placed in a prone position with mild sedation and local anesthesia. First, fine 20-gauge spinal needles we placed to mark the pedicles at T12 and L1. Next from the left side, away from the vertical fracture 2 cc of non-ionic contrast were injected with a 22-gauge needle into the T12-L1 disc space to document if it communicated with the vertical fracture. Under fluoroscopy, the contrast could be seen passing form the disc space into the vertical fracture line of T12 establishing the fracture and disc were connected. Second, an 11-gauge vertebroplasty cannula was introduced into the left T12-L1 disc space (Figure 3A). A fine curette was passed through the cannula to the T12-L1 disc space to further open the communication (Figure 3B) and then 1.4 cc of Cortoss cement was injected, which slowly flowed from the disc space into the base of the fracture (Figure 3C, 3D). Third, to guarantee filling of the vertical part of the fracture on the right side, another 11-gauge vertebroplasty cannula was introduced through the pedicle of T12 and advanced under fluoroscopy until the fracture line was encountered (Figure 3E). The softness and gap of the fracture could be clearly felt and then a bone drill and curette were passed to open the space allowing another 1.4 cc of Cortoss to be injected into the vertical fracture (Figure 3F). Finally a cannula was introduced into the L1-L2 interspace where there was additional osteonecrotic changes and an additional 1.4 cc of Cortoss cement was injected to L1-L2. 

**Figure 4:**
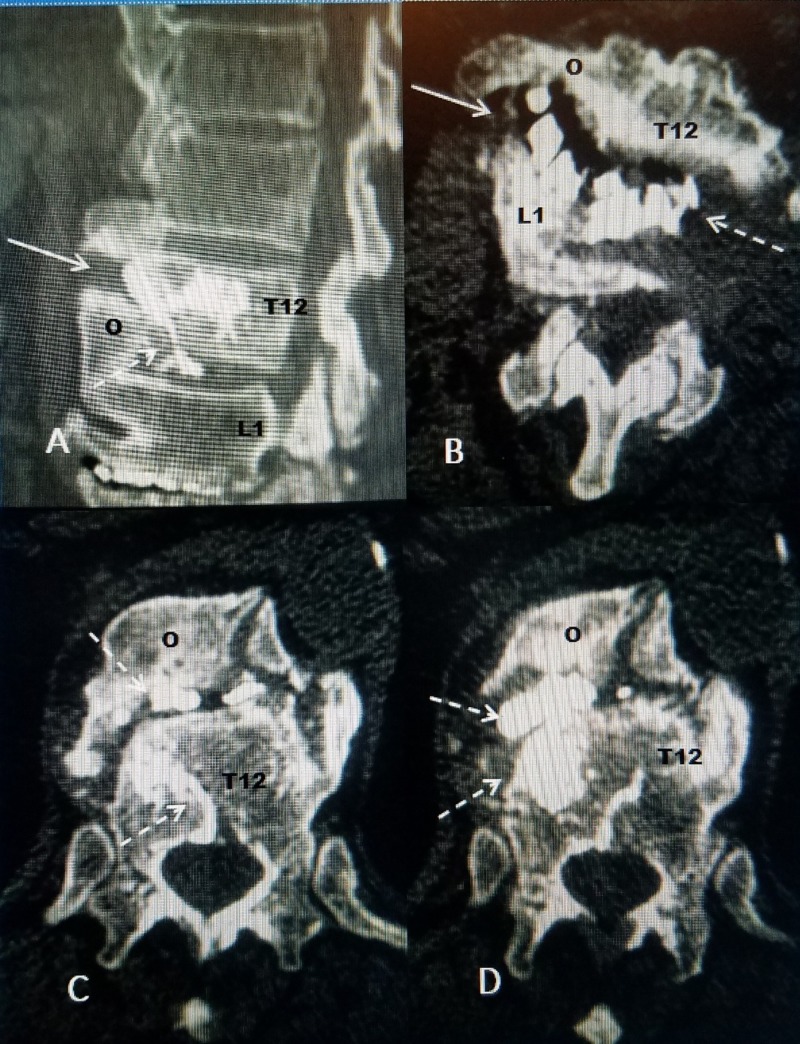
Follow-up CT scans A: Sagittal CT scan showing cement (white) within vertical avulsion fracture of anterior inferior part of T12 (solid white arrow). The T12 osteophyte (o) is seen to be fused and bridging to L1. B: Axial CT scan shows the cement within the anterior and lateral part of the fracture (dashed white arrow). This is connected but in a slightly different location than interspace cement (dashed white arrow) C: Axial CT showing the osteophyte (o) with cement scattered within the fracture (dashed white arrows). D: Axial CT showing cement (dashed white arrows) behind the osteophyte (o) of L1 and also within the more middle and lateral part of the adjacent vertebral body of T12.

The patient tolerated the procedure without problems and noted a significant decrease in the pain level in the recovery room. The three-month and six-month follow-up has continued to demonstrate resolution of the upper lumbar and lower thoracic pain with resumption of his activities. A follow-up CT scan was performed three months after the procedure to document exactly the position of the bone cement relative to the fracture and the progressive vacuum changes both in the T12-L1 disc space and the vertical avulsion fracture. The follow-up CT scan shows there is scattered cement placement both in the fracture and the T12-L1 disc space but the large vacuum cleft and especially the tract connecting the spaces are filled with cement (Figure 4).

## Discussion

This case presents an unusual combination of a vertical avulsion type fracture with very localized pain, developing in an area of vertebral instability above a previous L2 to S1 instrumented long lumbar fusion. There were also large ventrally bridging osteophytes from T9 to L1 and ossification of the posterior supraspinous ligaments resulting in more rigidity of the thoraco-lumbar spine. The patient developed a vertical fracture of the inferior endplate and adjoining osteophyte at T12 that connected to the T12-L1 disc space. The patient started steadily developing worsening upper lumbar and lower thoracic pain after undergoing implantation of a spinal cord stimulator. It is unclear if positioning during surgery may have contributed to the development of the fracture which can be seen in patients with DISH [2-3]. CT scans over 21 months revealed worsening osteonecrosis indicated by CT vacuum changes in the fracture and the communicating disc space indicative of instability both in the intra-discal space as well as along the fracture line in the non-fused bridging area. Spinal avulsion fractures typically involve the anterior and inferior part of the endplate due to hyperextension [4]. The patient also had previous chemotherapy and multiple steroid injections making him at higher risk for vertebral osteoporosis [2-3].

Spinal rigidity has multiple etiologies including previous multilevel lumbar fusions, as in this patient, from L2 to L5. The extensive ventral bridging and resultant rigidity from large spinal osteophytes are also characteristic of DISH [2-3]. DISH is radiologically defined when there are at least four contiguous segments of bridging osteophytes as in this patient. These patients are vulnerable to spinal fractures with no or minor trauma, or even positioning during general surgery [2, 5]. Avulsion fractures are typically vertical and involve the anterior vertebral apophyseal ring and can be associated with spinal hyperextension injury, leading to avulsion of the anterior spinal ligament [4]. In this case, the vertical fracture included the anterior and inferior edge of the T12 vertebral body and the edge of the osteophyte. There were additional bridging osteophytes at L1-L2 and posterior ossification along the supraspinous ligament.

Vacuum changes either on CT or MRI scans have been noted as a sign of instability and osteonecrosis commonly seen with thoraco-lumbar osteoporotic compression fractures. Clefts identified with vacuum changes are shown to change size with flexion and extension reflecting the underlying micro-instability with the fracture. Filling of the cleft with cement at the time of vertebroplasty is felt to be critical to resolving the symptoms in these patients [6-7]. Vacuum changes within the disc space or facet joints are also noted with spinal instability [7-8]. In this case, the communication of the intra-discal vacuum change at T12-L1 with the vacuum change seen in vertical anterior third avulsion fracture of T12 is unusual. After documenting the communication of the disc space to the fracture with discography and then by placing multiple cannulas in different positions to ensure filling of the fracture defects it was possible to fill the majority of the larger cleft areas with cement.

Treatment of fractures with DISH is complicated by the patient’s age and often associated osteoporosis. Besides bracing, fusion with instrumentation is recommended [2, 9]. This elderly patient, who already had two spinal instrumentations extending the fusion to L2, did not want to consider further open surgery or additional spinal instrumentation. Besides treating osteoporotic fractures, percutaneous vertebral augmentation has been used to stabilize loose pedicle screws and non-fused and loosened inter-body implants [10]. This case demonstrates that accurate placement of cannulas and then cement, carefully injected along the majority of the fracture, including the interspace that in this patient communicated with the fracture may be a simple, minimal procedure to stabilize the patient and relieve pain. Studies of vacuum changes have shown that, when using bone cement, it is critical to make sure the area of osteonecrosis and vacuum change is filled with the cement. 

## Conclusions

Spinal rigidity, either due to DISH, anterior multilevel osteophytes, or previous spinal fusion in elderly patients can be associated with osteoporotic spinal fractures and localized instability. Findings of increasing vacuum signal change on either CT or MRI scans, regarded as a clear radiologic indication of osteonecrosis and instability, concurrent with worsening and localized pain, can be an indication of clinical symptomatic segmental instability. Multilevel fusion and instrumentation to stabilize these difficult fractures have been the recommended treatments, but in certain cases, percutaneous vertebral augmentation may be a reasonable alternative approach to treatment. When there are large radiologic vertebral clefts, it is critical to get the cement into the cleft to get symptomatic relief and prevent further fracture progression. This has been shown to be an effective treatment with osteoporotic fractures as well as in cases with instability due to loose pedicle screws and poorly incorporated interbody grafts. In this case, when contrast injected into the involved disc space documented the communication with the adjacent avulsion fracture, the unstable segment was able to be treated with percutaneous multi-site injections of bone cement with lasting symptomatic pain relief.
